# PI3Kγ kinase activity is required for optimal T-cell activation and differentiation

**DOI:** 10.1002/eji.201343812

**Published:** 2013-10-09

**Authors:** Nadia Ladygina, Sridevi Gottipati, Karen Ngo, Glenda Castro, Jing-Ying Ma, Homayon Banie, Tadimeti S Rao, Wai-Ping Fung-Leung

**Affiliations:** Janssen Research & Development, LLCSan Diego, CA, USA

**Keywords:** Cell activation, Cell differentiation, Immune responses, PI3K gamma, T cells

## Abstract

Phosphatidylinositol-3-kinase gamma (PI3Kγ) is a leukocyte-specific lipid kinase with signaling function downstream of G protein-coupled receptors to regulate cell trafficking, but its role in T cells remains unclear. To investigate the requirement of PI3Kγ kinase activity in T-cell function, we studied T cells from PI3Kγ kinase-dead knock-in (PI3Kγ^KD/KD^) mice expressing the kinase-inactive PI3Kγ protein. We show that CD4^+^ and CD8^+^ T cells from PI3Kγ^KD/KD^ mice exhibit impaired TCR/CD28-mediated activation that could not be rescued by exogenous IL-2. The defects in proliferation and cytokine production were also evident in naïve and memory T cells. Analysis of signaling events in activated PI3Kγ^KD/KD^ T cells revealed a reduction in phosphorylation of protein kinase B (AKT) and ERK1/2, a decrease in lipid raft formation, and a delay in cell cycle progression. Furthermore, PI3Kγ^KD/KD^ CD4^+^ T cells displayed compromised differentiation toward Th1, Th2, Th17, and induced Treg cells. PI3Kγ^KD/KD^ mice also exhibited an impaired response to immunization and a reduced delayed-type hypersensitivity to Ag challenge. These findings indicate that PI3Kγ kinase activity is required for optimal T-cell activation and differentiation, as well as for mounting an efficient T cell-mediated immune response. The results suggest that PI3Kγ kinase inhibitors could be beneficial in reducing the undesirable immune response in autoimmune diseases.

Additional supporting information may be found in the online version of this article at the publisher's web-site

## Introduction

Phosphatidylinositol-3-kinase gamma (PI3Kγ) is a member of the PI3K family that phosphorylates phosphatidylinositol 4,5-diphosphate to generate phosphatidylinositol 3,4,5-triphosphate (PIP3) at the plasma membrane 1. PIP3 serves as a docking station to recruit signaling proteins containing the pleckstrin homology domain for initiation of signaling events 2. PI3K family can be categorized into class I, II, and III, whereas class I PI3K is further divided into class IA and IB subsets 1,3. Class IA PI3K consists of three members PI3Kα, PI3Kβ, and PI3Kδ. PI3Kγ is the only member in the class IB subset and it is a heterodimer composed of a catalytic subunit p110γ and one of the two regulatory subunits p101 and p84. PI3Kγ is involved in G protein-coupled receptor (GPCR) signaling through interaction with G protein subunit G_βγ_ 4,5. Although low levels of PI3Kγ have been found in cardiomyocytes, PI3Kγ expression is otherwise restricted to the hematopoietic lineage, suggesting its functional importance in leukocytes 6,7.

PI3Kγ is involved in T-cell development in the thymus but its role in T-cell activation has been controversial 8. Although T cells from PI3Kγ-deficient mice have been reported to be defective in proliferation and cytokine production 8–10, other studies with independently generated PI3Kγ-deficient mice demonstrated a normal T-cell proliferative response 11,12. A number of PI3Kγ kinase inhibitors have been shown to block T-cell functions but interpretation of PI3Kγ biology from compound effects is limited by the target selectivity of compounds 13. Further investigations of PI3Kγ function in T cells with alternative approaches are therefore warranted.

Upon TCR engagement, multiple signaling mechanisms including NFAT, NF-κB, and MAPK pathways are activated, which result in gene induction and cell cycle progression. Generation of PIP3 is one of the earliest signals observed in activated T cells 14,15. Class IA PI3K members could be recruited to TCR complex via their regulatory subunits 16–19. Costimulatory receptors CD28 and ICOS have class IA PI3K binding motifs YXXM on their cytoplasmic domains 20,21. Class IA PI3K activity in T cells has also been suggested to be downregulated by PIK3IP1 22. Protein kinase B (AKT) is a serine threonine kinase downstream of PI3K and it has been reported to modulate NF-κB signaling pathway during T-cell activation 23. Although activated T cells from PI3Kγ-deficient mice show a reduced phosphorylation of AKT and ERK, details on how PI3Kγ is recruited in TCR signaling remain unclear 9.

PI3K has been shown to play a role in T-cell differentiation through TORC1/TORC2 signaling pathways and transcription factors Forkhead box (FOXO) and Krueppel-like factor 2 (KLF2) 24,25. Expression of Th17 cytokine IL-17A by human CCR6^+^ CD4^+^ T cells can be induced by IL7 and this induction was blocked by PI3K inhibitors 24. Recently a PI3Kγ inhibitor was reported to block Th17 differentiation in human CD4^+^ T cells 26. PI3Kγ-deficient mice were also shown to be protected in a Th17 cell-driven psoriasis model 10. However, the potential role of PI3Kγ in T-cell polarization to different helper T-cell subsets or regulatory T (Treg) cells has not been studied in details.

The scaffolding function of PI3Kγ independent of its kinase activity has been demonstrated in the cardiovascular system 7. However, the majority of reports on the role of PI3Kγ in immune cells have been based on studies of PI3Kγ^-^deficient mice 8,27. Defects identified from this approach cannot differentiate the biological role of PI3Kγ coming from its kinase activity or adaptor function. Mice defective in PI3Kγ kinase activity (PI3Kγ kinase-dead knock-in (PI3Kγ^KD/KD^)) have been generated by introducing a point mutation into the p110γ gene 7. In this report, we studied the response of T cells from this mouse line and show that PI3Kγ kinase activity is required for optimal T-cell activation and differentiation. Defects in T-cell response are also observed in immunization and DTH models. These results demonstrate a kinase-dependent role of PI3Kγ in T-cell function and suggest that PI3Kγ could be a target of interest in drug discovery for treatment of inflammation and autoimmune diseases. PI3Kγ kinase inhibitors could, therefore, be beneficial in treatment of T cell-mediated autoimmune and inflammatory diseases.

## Results

### Defective TCR-mediated activation of PI3Kγ^KD/KD^ T cells

**Figure 1:**
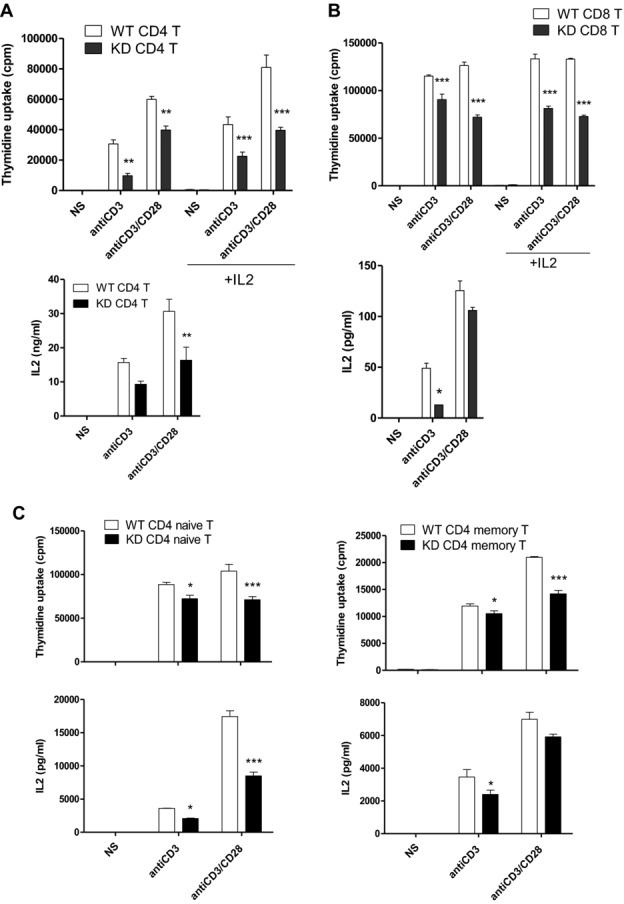
Defective activation of PI3Kγ^KD/KD^ T cells. (A) CD4^+^ T cells, (B) CD8^+^ T cells, and (C) CD4 naïve and memory T cells from WT and PI3Kγ^KD/KD^ (KD) mice were either not stimulated (NS) or stimulated with immobilized anti-CD3 (1 μg/mL) alone or in combination with soluble anti-CD28 (1 μg/mL) for 2 days in the presence or absence of exogenous IL-2 (100 U/mL). (A–C) T-cell proliferation and secreted IL-2 data are shown as mean + SEM of *n* = 3, and are representative of two independent experiments. *p < 0.05, **p < 0.01, ***p < 0.001; two-way ANOVA test.

To understand the role of PI3Kγ kinase activity in T-cell response, we studied T cells from PI3Kγ^KD/KD^ mice in different activation conditions. Expression of the kinase-inactive PI3Kγ protein in CD4^+^ T cells was comparable to wild-type (WT) T cells (Supporting Information Fig. 1). Proliferations of PI3Kγ^KD/KD^ CD4^+^ T cells upon anti-CD3 and anti-CD3/CD28 stimulations were reduced by 68 and 34%, respectively, compared to WT CD4^+^ T cells (Fig.1A). These cells produced less IL-2 and addition of exogenous IL-2 did not restore their proliferation to normal levels. Similar activation defects were demonstrated in PI3Kγ^KD/KD^ CD8^+^T cells as well (Fig.1B). Naïve and memory CD4^+^ T cells from PI3Kγ^KD/KD^ mice were tested for their requirement of PI3Kγ kinase activity in activation. Both naïve and memory cells showed a reduction in proliferation and cytokine production regardless of their Ag preexposure histories (Fig.1C).

### Impaired mixed lymphocyte reaction (MLR) and Ag-specific activation of PI3Kγ^KD/KD^ T cells

**Figure 2:**
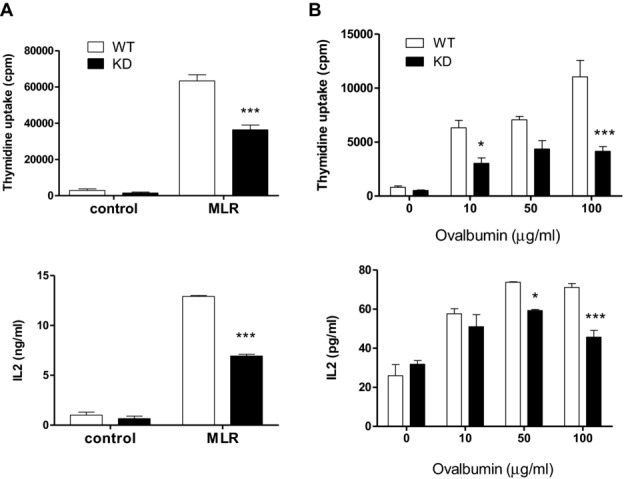
Impaired Ag-specific activation of PI3Kγ^KD/KD^ T cells. (A) CD4^+^ T cells from from WT and PI3Kγ^KD/KD^ (KD) mice of C57BL/6 genetic background responded to allogeneic BALB/c splenocytes in a 3-day MLR. (B) Enriched ovalbumin-specific CD4 effector T cells derived from WT and KD mice responded to ovalbumin in a 3-day stimulation. T-cell proliferation and secreted IL-2 data are shown as mean + SEM of *n* = 3 and are representative of two independent experiments. *p < 0.05; ***p < 0.001; two-way ANOVA test.

The requirement of PI3Kγ kinase activity in T-cell activation was further examined in Ag-specific stimulations. In MLRs, CD4^+^ T cells from WT and PI3Kγ^KD/KD^ mice of C57BL/6 genetic background were stimulated with allogeneic BALB/c splenocytes. The allogeneic response mounted by PI3Kγ^KD/KD^ CD4^+^ T cells was significantly less than WT CD4^+^ T cells, with a 35% decrease in proliferation and IL-2 production (Fig.2A).

To evaluate T-cell response to specific Ags, ovalbumin-specific effector T cells were generated from CD4 T cells of ovalbumin-immunized WT and PI3Kγ^KD/KD^ mice after multiple rounds of in vitro ovalbumin restimulation. An ovalbumin dose-dependent recall response was demonstrated in these T cells and the proliferative response of PI3Kγ^KD/KD^ T cells was reduced by 38 to 62% accompanied with a decreased IL-2 production compared to WT T cells (Fig.2B). Taken together, we have demonstrated the requirement of PI3Kγ kinase activity for optimal Ag-specific T-cell activation.

### Mechanism of reduced activation of PI3Kγ^KD/KD^ T cells

**Figure 3:**
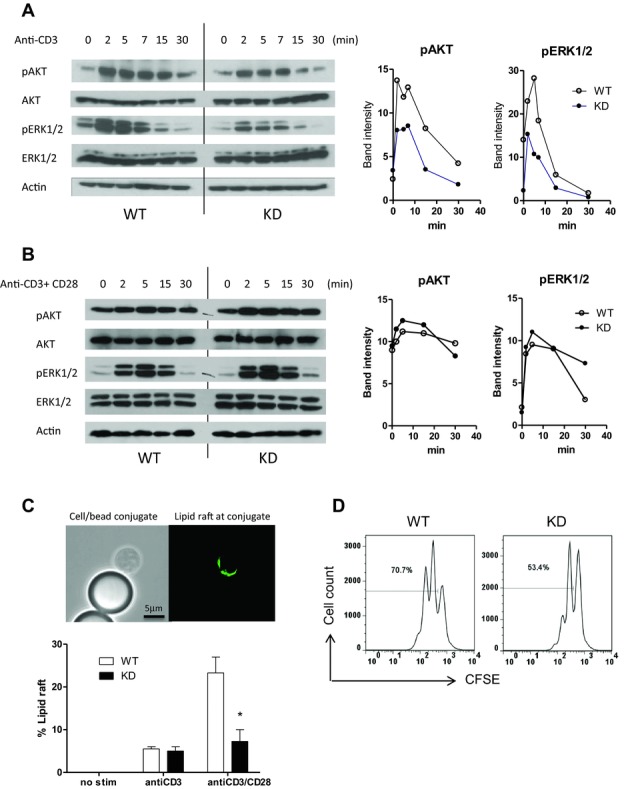
Mechanistic analysis of PI3Kγ^KD/KD^ T-cell activation. The kinetics of AKT and ERK phosphorylation in WT and PI3Kγ^KD/KD^ (KD) CD4^+^ T cells upon stimulation with (A) anti-CD3 alone or (B) anti-CD3/CD28 is shown in immuno-blots, and signals were quantitated and plotted as band intensity versus time in graphs. (C) Lipid rafting formation on T cells at contact areas with anti-CD3- or anti-CD3/CD28-coated beads were detected by FITC-cholera toxin B under fluorescent microscope. Percentages of cell/bead conjugates with lipid raft formation are shown as mean + SEM of *n* = 2. *p < 0.05; two-way ANOVA test. (D) Cell division of CFSE-stained CD4^+^ T cells after 3 days of anti-CD3/CD28 stimulation was analyzed by FACS and percentage of CFSE^low^ divided cells was shown in histograms. (A–D) Data are representative results of two and three independent experiments.

The mechanism of PI3Kγ involvement in T-cell response was investigated in a series of studies to monitor the early downstream events of T-cell activation. Upon anti-CD3 stimulation, phosphorylation of AKT and ERK1/2 in PI3Kγ^KD/KD^ T cells was reduced although the induction kinetics was normal (Fig.3A). The peak levels of phosphorylated AKT and ERK1/2 in PI3Kγ^KD/KD^ T cells decreased by 34 and 62%, respectively, compared to WT T cells. These phosphorylation defects, however, were overcome by stimulation with anti-CD3/CD28, possibly due to recruitment of other PI3K members of the class IA family (Fig.3B).

In the process of T-cell activation, lipid rafts on T cell are accumulated at the contact area with APC 28. Anti-CD3- and anti-CD28-coated polystyrene beads can mimic APC effect in initiating lipid raft aggregation on T cells, which can be detected with FITC-conjugated cholera toxin B (Fig.3B). Lipid raft formation on PI3Kγ^KD/KD^ T cells was 70% reduced when compared to WT T cells (Fig.3C).

T-cell activation eventually leads to cell cycle progression and the kinetics of cell division was monitored in CFSE-stained T cells. PI3Kγ^KD/KD^ T cells were slower than WT T cells in cell division, with 53% of divided PI3Kγ^KD/KD^ T cells, which were shown as CFSE^low^ cells after 5 days of stimulation, a significant reduction when compared to 70% of divided WT T cells (Fig.3D).

### Reduced differentiation of PI3Kγ^KD/KD^ T cells to Th1, Th2, Th17, and Treg cells

**Figure 4:**
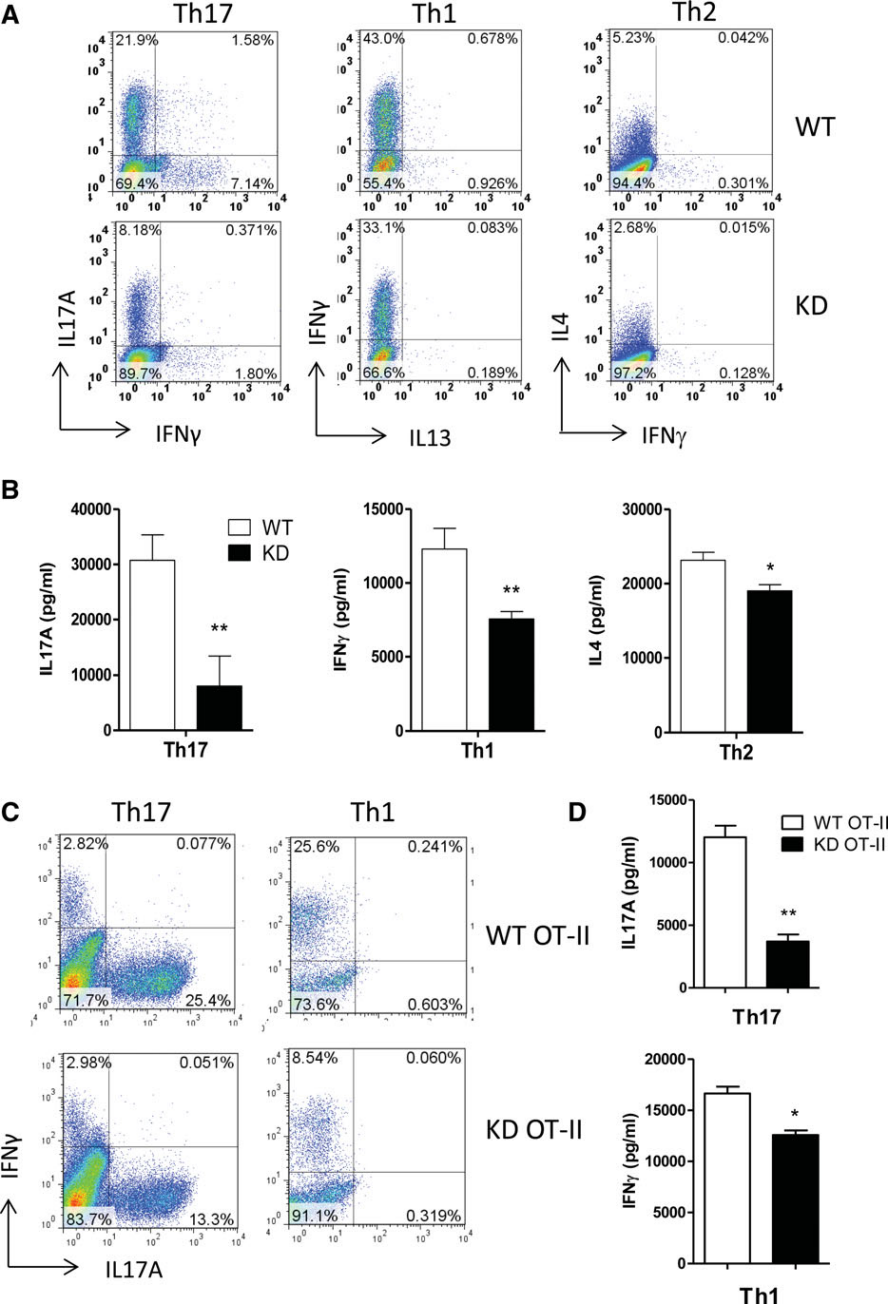
Reduced differentiation of PI3Kγ^KD/KD^ T cells to Th17, Th1, and Th2 cells. Naïve CD4^+^ T cells from WT and PI3Kγ^KD/KD^ (KD) mice were stimulated with anti-CD3/CD28 under different polarization conditions for 6 days. (A) Percentages of IL-17A expressing Th17 cells, IFN-γ expressing Th1 cells, and IL-4 expressing Th2 cells were shown in FACS plots. (B) Day 6 differentiated T cells were restimulated overnight with anti-CD3/CD28 in plain culture medium and secreted cytokines were measured. (C) CD4^+^ T cells from WT and PI3Kγ^KD/KD^ (KD) mice expressing the OT-II transgenic TCR were activated with the OVA 323–339 peptide for 6 days in the presence of mitomycin C-treated splenocytes under Th1 or Th17 polarization condition. Percentages of IL-17A expressing Th17 cells and IFN-γ expressing Th1 cells were shown in FACS plots. (D) Secreted cytokines from day 6 differentiated transgenic T cells were measured after overnight stimulation with anti-CD3/CD28 in plain culture medium. Secreted cytokine data are shown as mean + SEM of *n* = 3. **p < 0.01; two-tailed Student's *t*-test. (A–D) Data shown are representative of one of two independent experiments performed.

To explore the role of PI3Kγ kinase activity in helper T-cell differentiation, naïve CD4^+^ T cells from WT and PI3Kγ^KD/KD^ mice were stimulated with anti-CD3/CD28 antibodies in the presence of differentiating cytokines to drive T-cell polarization to Th1, Th2, or Th17 cells. After 6 days of culture, differentiated T cells were identified by distinct intracellular cytokines in flow cytometry following the cell gating strategy shown in Supporting Information Figure 2. PI3Kγ^KD/KD^ T cells were less effective in polarization to different helper T cells in particular Th17 cells (Fig.4A). There were 8% of IL-17A^+^ cells in PI3Kγ^KD/KD^ Th17 cultures compared to 22% in WT cultures, demonstrating a 64% decrease of Th17 differentiation from PI3Kγ^KD/KD^ T cells. Differentiation of PI3Kγ^KD/KD^ T cells to Th1 cells was reduced by 23%, with 33% of IFN-γ^+^ cells detected in PI3Kγ^KD/KD^ cultures compared to 43% in WT cells. Similar defect was also observed in Th2 differentiation, with 3% of IL-4^+^ cells in PI3Kγ^KD/KD^ Th2 cultures compared to 5% in WT cells. Measurement of cytokines secreted by differentiated helper T cells confirmed the flow cytometry findings (Fig.4B). The level of IL-17A in PI3Kγ^KD/KD^ Th17 cultures was reduced by 74% compared to WT cultures. IFN-γ in PI3Kγ^KD/KD^ Th1 cultures was reduced by 38%, whereas IL-4 in PI3Kγ^KD/KD^ Th2 cultures was reduced by 18%.

Differentiation of PI3Kγ^KD/KD^ T cells was further investigated in Ag-specific stimulations. To obtain Ag-specific T cells that have not been differentiated to specific helper cell types previously, we purified naïve CD4^+^ T cells from WT and PI3Kγ^KD/KD^ mice expressing the ovalbumin-specific transgenic OT-II TCR. These T cells were stimulated with ovalbumin peptide OVA 323–339 in the presence of C57BL/6 splenocytes as APC, as well as differentiating cytokines to drive Th1 or Th17 polarization. A profound activation of transgenic T cells was initiated and a significant number of differentiated T cells were generated after 6 days of culture. Differentiation of PI3Kγ^KD/KD^ OT-II T cells to Th1 and Th17 cells were reduced by 48 and 67%, respectively, when compared to WT OT-II T cells (Fig.4C). There were 13% of IL-17A^+^ cells in PI3Kγ^KD/KD^ Th17 cultures, compared to 25% in WT cultures. Under Th1 differentiation condition, PI3Kγ^KD/KD^ T cells gave rise to 8% of IFN-γ^+^ cells compared to 25% in WT cells. Consistent with the flow cytometry results, the level of IL-17A in PI3Kγ^KD/KD^ Th17 culture was reduced by 69%, and IFN-γ in PI3Kγ^KD/KD^ Th1 culture was reduced by 24% compared to WT cultures (Fig.4D).

**Figure 5:**
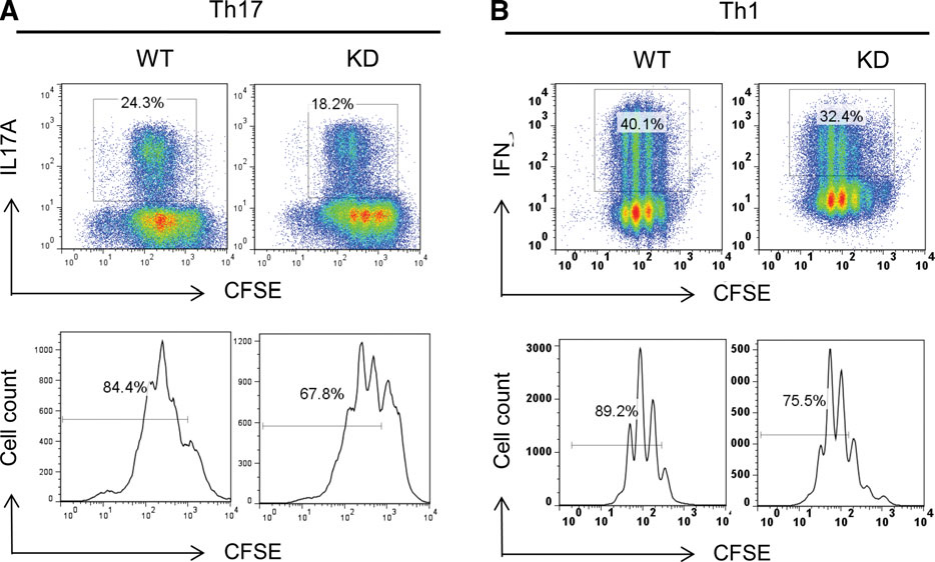
Correlation of PI3Kγ^KD/KD^ T-cell defects in differentiation and cell division. Naïve CD4^+^ T cells from WT and PI3Kγ^KD/KD^ (KD) mice were stained with CFSE and stimulated with anti-CD3/CD28 under (A) Th17 or (B) Th1 polarization condition for 5 days. Day 5 differentiated cells were analyzed in flow cytometry to identify Th17 and Th1 cells by intracellular expression of IL-17A and IFN-γ, respectively, and cell division was monitored by the decrease in CFSE fluorescent signals in histograms and percentages of CFSE^low^ divided cells were indicated. (A and B) Data shown are representative of one of two independent experiments performed.

To understand the mechanism of defective helper cell differentiation from PI3Kγ^KD/KD^ T cells, we stained T cells with CFSE and studied the correlation of T-cell differentiation with cell division. Differentiation of PI3Kγ^KD/KD^ T cells to Th17 cells was reduced by 25%, with 18% IL-17A^+^ cells in the PI3Kγ^KD/KD^ cultures compared to 24% in the WT cultures (Fig.5A). Division of PI3Kγ^KD/KD^ T cells in the same cultures was reduced by 20%, with 68% divided cells detected in PI3Kγ^KD/KD^ cultures compared to 84% in WT cells (Fig.5A). Under Th1 differentiation condition, PI3Kγ^KD/KD^ Th1 cells were reduced by 19%, with 32% IFN-γ^+^ cells in the PI3Kγ^KD/KD^ cultures and 40% in the WT cultures (Fig.5B). Division of PI3Kγ^KD/KD^ T cells in the corresponding cultures was reduced by 15%, with 75% divided cells in PI3Kγ^KD/KD^ cultures compared to 89% in WT cells (Fig.5B). Taken together, the decrease in PI3Kγ^KD/KD^ T-cell differentiation correlated closely with the delay in cell cycle progression.

**Figure 6:**
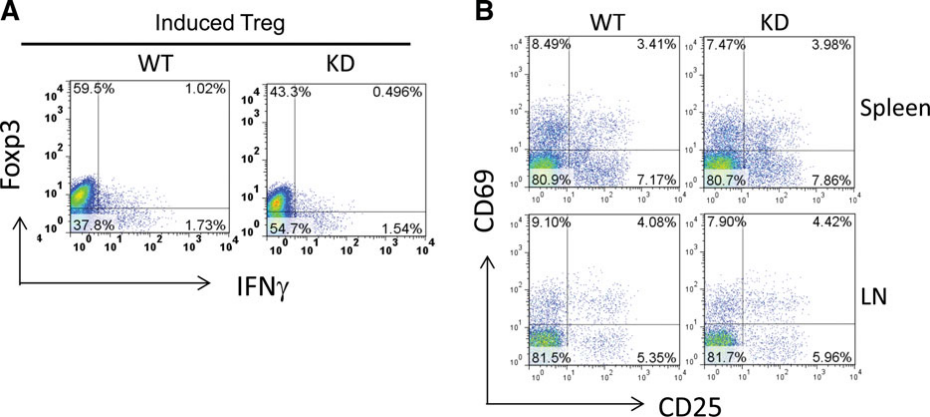
Reduced differentiation of PI3Kγ^KD/KD^ CD4 T cells to induced Treg cells but normal thymus-derived natural Treg cells in PI3Kγ^KD/KD^ mice. (A) Induced Treg cells were differentiated from WT and PI3Kγ^KD/KD^ (KD) naïve CD4^+^ T cells after 6 days of stimulation with anti-CD3/CD28 under Treg differentiation condition as described in the Materials and methods. Induced Treg cells were identified by expression of transcription factor Foxp3 in FACS analysis. (B) Natural Treg cells in the spleens and inguinal lymph nodes (LN) of WT and KD mice were identified as CD25^+^ CD69^−^ cells in the gated CD4^+^ T-cell populations. (A and B) Data shown are representative of one of two independent experiments performed.

Differentiation of PI3Kγ^KD/KD^ T cells to Treg cells was studied by activating naïve CD4^+^ T cells with anti-CD3/CD28 antibodies in the presence of TGF-β1 and IL-2. A moderate 27% reduction in induced Treg-cell differentiation was demonstrated in PI3Kγ^KD/KD^ T cells (Fig.6A). There were 43% of Foxp3^+^ induced Treg cells generated from PI3Kγ^KD/KD^ T cells, compared to 59% from WT T cells. In contrast to the moderate defect in induced Treg differentiation, thymus-derived natural Treg cells in PI3Kγ^KD/KD^ mice appeared to be normal and the populations of natural Treg cells identified as CD25^+^ CD69^−^ CD4^+^ T cells in the spleen and lymph nodes were comparable to those in WT mice (Fig.6B).

### Decreased chemotaxis of PI3Kγ^KD/KD^ T cells toward chemokines

**Figure 7:**
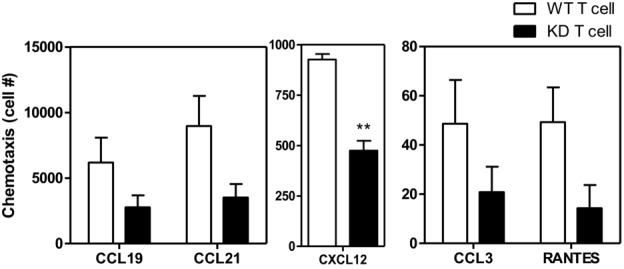
Chemotactic defects of PI3Kγ^KD/KD^ T cells. Splenocytes from WT and PI3Kγ^KD/KD^ (KD) mice were used in the transwell chemotaxis assays. Chemotaxis of T cells toward chemokines CCL19, CCL21, CXCL12, CCL3, and RANTES was measured as described in the Materials and methods. Data are shown as mean + SEM of *n* = 3 and are representative of two independent experiments. **p < 0.01; two-tailed Student's *t*-test.

The requirement of PI3Kγ kinase activity in CD4^+^ T-cell chemotaxis was examined in the transwell migration assay. Chemotaxis of PI3Kγ^KD/KD^ T cells toward chemokines CCL3, CCL19, CCL21, CXCL12, and RANTES was reduced by 55 to 70% compared to WT T cells (Fig.7). Expression of the corresponding chemokine receptors on PI3Kγ^KD/KD^ T cells was found to be at normal levels (unpublished data). The results suggest a functional defect in chemotactic response of PI3Kγ^KD/KD^ T cells.

### Defective immune response in PI3Kγ^KD/KD^ mice

**Figure 8:**
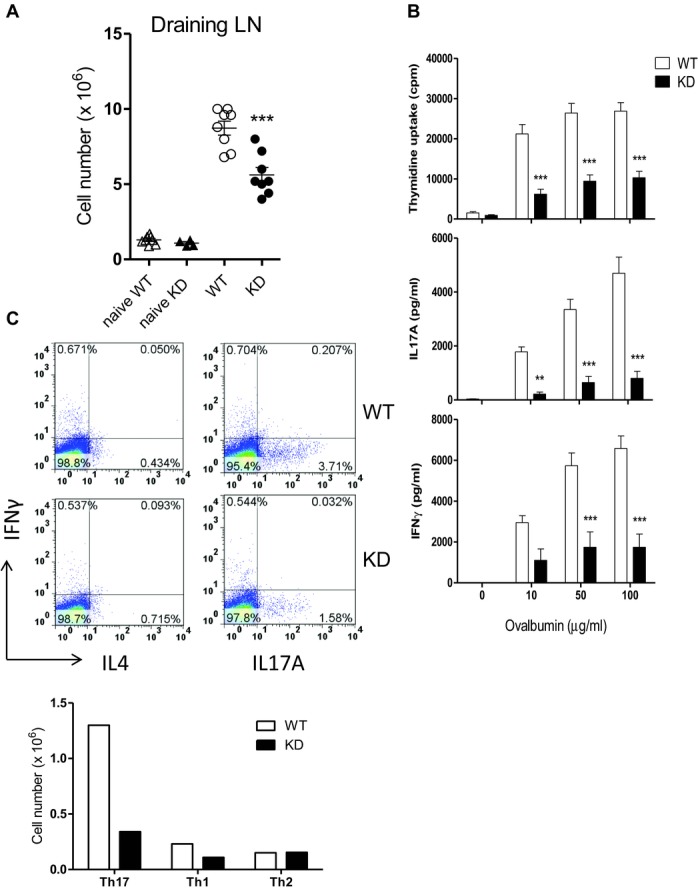
Impaired immune response of PI3Kγ^KD/KD^ mice to OVA/CFA immunization. WT and PI3Kγ^KD/KD^ (KD) mice (eight mice per group) were immunized with ovalbumin/CFA and mice injected with saline (naïve) were used as negative controls. Ten days after immunization, mice were sacrificed and inguinal lymph nodes were collected. (A) Cellularity of draining lymph nodes was quantitated. (B) Proliferation and cytokine production from draining lymph node cells after 3-day ex vivo stimulation with ovalbumin were measured. (C) Percentages of Th17, Th1, and Th2 cells in the draining lymph node cells were identified by intracellular cytokines IL-17A, IFN-γ, and IL-4, respectively in FACS analysis. Cell numbers of different helper T-cell subsets were calculated from the lymph node cell number and the percentage of helper cell subsets. (A–C) Data are shown as mean + SEM and are representative of one of two independent experiments performed. **p < 0.01;***p < 0.001; two-way ANOVA.

Our in vitro studies showed that T cells from PI3Kγ^KD/KD^ mice were less effective in activation, differentiation, and chemotaxis. The physiological importance of PI3Kγ kinase activity was further investigated in two immunization models. PI3Kγ^KD/KD^ and WT mice were immunized with ovalbumin/CFA at the tail base and the immune response in mice was examined 10 days later. The typical response in immunized mice is an enlargement of draining lymph nodes with increased cellularity as a result of expansion of Ag-specific T and B cells. Although lymph nodes from PI3Kγ^KD/KD^ mice were enlarged compared to unimmunized mice, their cellularity was 36% less than that from WT immunized mice (Fig.8A). The presence of ovalbumin-specific T cells in the draining lymph nodes was demonstrated by a robust proliferation of lymph node cells to ovalbumin stimulation in a dose-dependent manner, whereas the proliferative response of PI3Kγ^KD/KD^ lymph node cells was reduced by 62 to 71% compared to WT cells (Fig.8B). This was accompanied by a decrease in cytokine production, with a reduction of 81 to 88% in IL-17A and 62 to 74% in IFN-γ levels (Fig.8B). Differentiated Th17, Th1, and Th2 CD4 T cells in the draining lymph nodes were identified by intracellular cytokines IL-17A, IFN-γ, and IL-4, respectively. Th17 and Th1 populations in PI3Kγ^KD/KD^ lymph nodes were reduced by 74 and 52% respectively, whereas the Th2 population was comparable to that in WT lymph nodes (Fig.8C).

**Figure 9:**
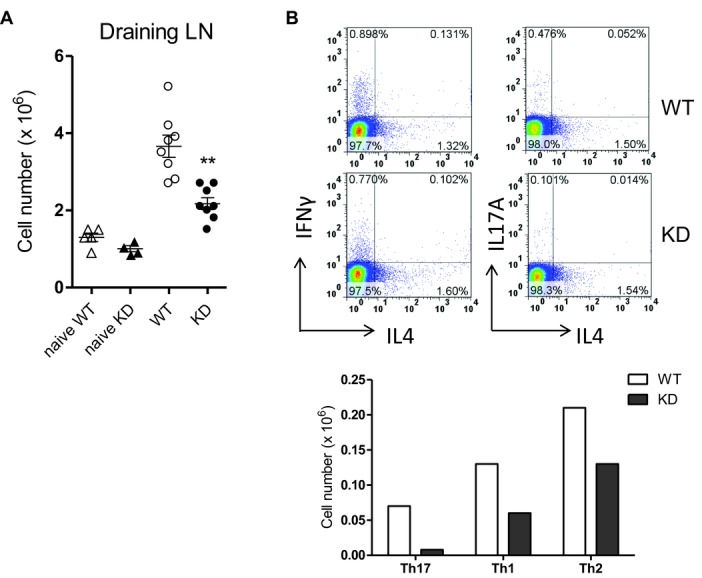
Impaired immune response of PI3Kγ^KD/KD^ mice to OVA/alum immunization. WT and PI3Kγ^KD/KD^ (KD) mice (eight mice per group) were immunized with ovalbumin/alum and mice injected with saline (naïve) were used as negative controls. (A) Cellularity of draining lymph nodes was quantitated and data are shown as mean + SEM of *n* = 8. **p < 0.01; two-way ANOVA test. (B) Th17, Th1, and Th2 CD4 T cells in draining lymph nodes were identify by intracellular cytokines IL-17A, IFN-γ, and IL-4 respectively. Cell numbers of different helper T-cell subsets were calculated from the lymph node cell number and the percentage of helper cell subsets. (A and B) Data are representative of one of two independent experiments performed.

PI3Kγ^KD/KD^ mice were further analyzed in a Th2 immunization model with ovalbumin/alum injection at the tail base and the immune response was analyzed 10 days later. PI3Kγ^KD/KD^ mice mounted an immune response with enlarged draining lymph nodes compared to unimmunized mice, but the cellularity of draining lymph nodes was reduced by 41% compared to that from WT immunized mice (Fig.9A). A significant Th2 cell population was generated in this model to allow analysis of Th2 differentiation in PI3Kγ^KD/KD^ mice. The IL-4^+^ Th2 population in the draining lymph nodes of PI3Kγ^KD/KD^ mice decreased by 38% compared to that in WT mice (Fig.9B). The IL-17A^+^ Th17 cells and IFN-γ^+^ Th1 cells in PI3Kγ^KD/KD^ lymph nodes were also reduced by 88 and 54%, respectively, when compared to WT lymph nodes. In summary, PI3Kγ kinase activity was shown to be needed for in vivo expansion and differentiation of Ag-specific T cells in response to immunization.

### Impaired DTH response in PI3Kγ^KD/KD^ mice

**Figure 10:**
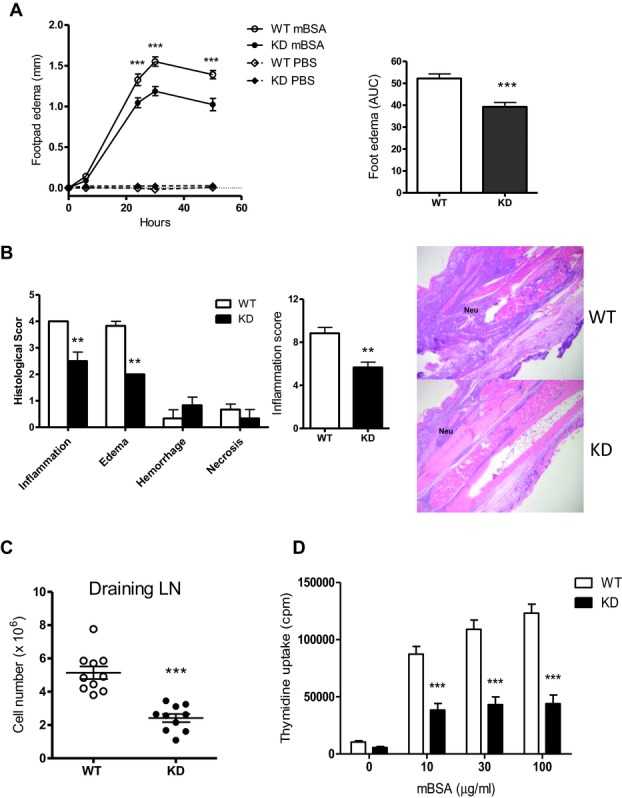
Reduced DTH response in PI3Kγ^KD/KD^ mice. WT and PI3Kγ^KD/KD^ (KD) mice (10 mice per group) were immunized with mBSA/CFA. Mice on day 7 after immunization were challenged with mBSA at left footpads and PBS at right footpads as negative controls. (A) Footpad thickness was measured at multiple time points and edema was defined as the increased thickness after footpad injection. The time course of edema and the area under curve values are shown. (B) Foot tissue sections were stained with H&E and infiltration of neutrophils (Neu) was indicated. Histological change and severity of tissue inflammation in sections were examined and scored on an arbitrary scale from 0 to 5 based on different criteria. Overall inflammation scores of WT and KD groups are also shown. (C) Cellularity of popliteal lymph nodes draining from footpad challenge sites was quantitated. (D) Proliferation of inguinal lymph node cells after 3-day ex vivo stimulation with mBSA was measured. (A–D) Data are shown as mean + SEM of *n* = 10 and are representative of one of two independent experiments performed. Statistical significance of histological scores were analyzed by Mann–Whitney test whereas other readouts were measured by two-way ANOVA test, and *p-*values were indicated as **p < 0.01 and ***p < 0.001.

PI3Kγ^KD/KD^ mice were further characterized in a DTH model to evaluate the importance of PI3Kγ kinase activity in T cell-mediated immune response to Ag reexposure. WT and PI3Kγ^KD/KD^ mice were immunized with methylated BSA (mBSA)/CFA at the tail base. Eleven days postimmunization, the animals were injected at the right hind paws with mBSA to elicit a DTH response, whereas the left hind paws were injected with saline as negative controls. The swelling of Ag-injected paws was measured at different time points up to 48 h postchallenge. PI3Kγ^KD/KD^ mice exhibited an impaired DTH response with a 25% reduction in edema by comparing the area under curve of edema scores with WT mice (Fig.10A). Histological examination of mBSA-injected paws revealed an overall 30 to 50% reduction in inflammation, edema, hemorrhage, and necrosis in the paws from PI3Kγ^KD/KD^ mice, compared to those from WT mice (Fig.10B). Histopathological examination of WT paws showed a markedly severe inflammation and edema from dorsal to ventral part of the paws. Multiple abscesses in the ventral part were also observed. The majority of infiltrated cells were neutrophils accompanied by small numbers of macrophages and lymphocytes. In contrast, the paws from PI3Kγ^KD/KD^ mice showed a moderate inflammation and edema mostly located in the dorsal part of the paws. There were fewer abscesses in the ventral part of the paw. Most of the infiltrated inflammatory cells were neutrophils and the cell numbers were reduced by 50% of that observed in WT paws (Fig.10B). The immune response to mBSA immunization was examined in draining inguinal lymph node cells. A 50% reduction in cellularity was observed in PI3Kγ^KD/KD^ lymph nodes when compared to WT lymph nodes (Fig.10C). Lymph node cells mounted an Ag-specific response to mBSA ex vivo stimulation in a dose-dependent fashion and the proliferative response of PI3Kγ^KD/KD^ lymph node cells was reduced by 56 to 64% when compared to WT cells (Fig.10D).

## Discussion

Despite the discovery of PI3Kγ as a unique member of the class I PI3K family coupled with distinct regulatory subunits for GPCR signaling in immune cells, the role of PI3Kγ in T cell-mediated immunity remains elusive. The function of PI3Kγ as a signaling protein involves its lipid kinase activity as well as its scaffolding function 1,7. Studies on PI3Kγ biology have largely been based on the functional defects shown in PI3Kγ knockout mice or with inhibitor treatments 8–10,26,29. These approaches have provided valuable information furthering the understanding of the biological functions of PI3Kγ; there are, however, limitations in interpretation of the results from these studies. The lack of PI3Kγ expression in knockout mice does not allow dissection of PI3Kγ kinase activity from its adaptor function. PI3Kγ inhibitors block kinase activity specifically and are, therefore, useful tools to demonstrate the importance of PI3Kγ kinase activity. However, due to the challenge in generating highly selective and potent PI3Kγ inhibitors, compound effects could be due to off-target activities. We therefore took the biological approach to use PI3Kγ^KD/KD^ mice to define the role of PI3Kγ kinase activity on T cells.

We examined the activation of PI3Kγ^KD/KD^ T cells with different stimuli including anti-CD3 alone or in combination with anti-CD28. The partial defects in T-cell proliferation and cytokine production could be demonstrated in both stimulations, and the defects could not be rescued with exogenous IL-2. The physiological relevance of this impairment was confirmed with Ag-specific stimulations in vitro and in vivo. In MLRs, the response of PI3Kγ^KD/KD^ T cells to allogeneic cells was reduced. PI3Kγ^KD/KD^ T cells from transgenic OT-II TCR mice and from ovalbumin-immunized mice were defective in initiating an optimal ovalbumin-specific response in vitro. Expansion of Ag-specific T cells in the draining lymph nodes of immunized PI3Kγ^KD/KD^ mice was also reduced. Interestingly, T-cell activation defects could not be overcome by increasing the strength of the stimuli, such as combination of anti-CD3 and anti-CD28 or high concentrations of Ags. Furthermore, defects could be shown in different T-cell subsets including CD4^+^ and CD8^+^ T cells, as well as naïve, memory, and effector T cells. Taken together, we have demonstrated here that PI3Kγ kinase activity is needed for optimal T-cell activation, although its role is not dominant and the lack of PI3Kγ kinase activity does not abolish T-cell activation completely.

The involvement of PI3Kγ kinase activity in T-cell polarization was explored in different stimulation conditions. Naïve PI3Kγ^KD/KD^ CD4^+^ T cells showed a reduced polarization to Th1, Th2, Th17, and induced Treg cells when stimulated with anti-CD3/anti-CD28 in the presence of differentiating cytokines. Defects in differentiation of Th1 and Th17 cells from Ag-specific stimulation were also confirmed using naïve PI3Kγ^KD/KD^ T cells expressing the OT-II transgenic TCR. The reduced differentiation of PI3Kγ^KD/KD^ T cells to Th or Treg cells could be the result of defective activation of PI3Kγ^KD/KD^ T cells. In vivo differentiation of PI3Kγ^KD/KD^ T cells was examined in two immunization models and reduced numbers of differentiated Th17, Th1, and Th2 cells were identified in the draining lymph nodes of immunized PI3Kγ^KD/KD^ mice. The decrease in Th17 cells seemed to be more significant than Th1 cells, whereas Th2 cells were affected minimally.

The defects demonstrated in PI3Kγ^KD/KD^ T cells are similar to the phenotype reported for PI3Kγ knockout mice 8–10, suggesting that the kinase activity could be the key function of PI3Kγ in T cells. This finding has an important implication to drug discovery since PI3Kγ inhibitors only block kinase activity but not adaptor function, and the results predict the potential efficacy of PI3Kγ inhibitors in the immune system. There is, however, a recent report suggesting an enhanced IL-17A production and Th17 differentiation from PI3Kγ deficient T cells 12. One possible explanation for the different findings is that our results are from T cells defective in PI3Kγ kinase activity whereas this report is based on T cells defective in PI3Kγ expression. Further studies are, therefore, needed to clarify the discrepancies.

The role of PI3Kγ kinase activity on T cells identified in our studies also resembles the biological effects of PI3Kδ 30,31. In fact, PI3Kγ and PI3Kδ are closely related in their biological properties. Both of them are expressed in cells of hematopoietic lineage, unlike the ubiquitous expression of other members PI3Kα and PI3Kβ. They are not dominant players in T-cell functions and the lack of their activities only reduces but does not abolish completely the response of T cells. Furthermore, their kinase activities are essential for their biological effects in T cells. Since they utilize different regulatory subunits for their signaling activities, it is possible that the role of PI3Kγ and PI3Kδ in TCR signaling does not completely overlap and dual target inhibition may achieve a better efficacy in blocking T-cell activation.

PI3Kγ signaling is known to be downstream of GPCR through interaction with G_βγ_ proteins 4,5. The mechanism of PI3Kγ involvement in TCR signaling is still largely unknown. It remains a key question as to whether PI3Kγ is directly involved in TCR signaling or indirectly through its participation in signaling of the GPCRs that function as TCR signaling mediators. Investigation of the defective mechanisms in PI3Kγ^KD/KD^ T cells revealed a decrease in TCR-mediated phosphorylation of AKT and ERK1/2, which could be compensated by TCR/CD28 stimulation, a defect in lipid raft aggregation, and a delay in cell cycle progression. It is possible that the lack of PI3Kγ kinase activity causes a defect in TCR signaling that results in a delay in cell cycle progression and eventually leads to a decrease in T-cell differentiation.

Here we have shown that PI3Kγ kinase activity contributes to T-cell activation, differentiation, and chemotaxis. Defects in T-cell response in vitro correlate with the reduced immune response in immunization and DTH models in PI3Kγ^KD/KD^ mice. These mice have normal natural Treg-cell population and do not seem to succumb to spontaneous autoimmune diseases, suggesting that Treg cells are functional for immune regulation in these mice. Indeed, PI3Kγ-deficient mice are also shown to be protected in a number of disease models including arthritis, psoriasis, lupus, colitis, experimental autoimmune encephalomyelitis, atherosclerosis, and asthmatic models 29,32–38. It should be noted that the in vivo results could potentially be partly coming from minor T-cell developmental defects observed in PI3Kγ^KD/KD^ and PI3Kγ-deficient mice 8 (our unpublished data). A definitive proof of PI3Kγ kinase activity on immune response in vivo may still rely on the availability of inducible PI3Kγ^KD/KD^ mouse models or highly selective PI3Kγ inhibitors. Our results with the PI3Kγ^KD/KD^ mice suggest a kinase-dependent role of PI3Kγ in T-cell immunity and support the notion that PI3Kγ kinase inhibitors could be beneficial in treatment of T cell-mediated autoimmune and inflammatory diseases. As we have demonstrated in this report, inhibition of PI3Kγ kinase activity but not its adaptor function could be an ideal scenario in drug discovery to achieve efficacy while avoiding possible cardiovascular liabilities. It is, therefore, imperative for both basic research and drug discovery to understand the molecular mechanism of PI3Kγ signaling and its functional role in the immune system.

## Materials and methods

### Mice

PI3Kγ^KD/KD^ mice were kindly provided by Dr. E. Hirsch (Molecular Biotechnology Center, University of Torino, Torino, Italy). PI3Kγ^KD/KD^ mice expressing the OT-II transgenic TCR were generated by cross-breeding OT-II transgenic mice (the Jackson Lab) with PI3Kγ^KD/KD^ mice in C57BL/6J genetic background. Mice were maintained under specific pathogen-free condition at the facility of Janssen Research & Development and the Jackson Lab. Mice were studied following protocols approved by the Institutional Animal Care and Use Committee of Janssen Research and Development.

### T-cell proliferation assays

CD8^+^ T cells, CD4^+^ T cells, and naïve or memory CD4^+^ T cells were isolated with Ab-coated magnetic bead kits (Miltenyi Biotech). T cells at 2 × 10^5^ cells/well were stimulated overnight with 1 μg/mL plate-bound anti-CD3 (Biolegend) with or without 1 μg/mL soluble anti-CD28 (Biolegend).

CD4^+^ T cells purified from splenocytes of ovalbumin/CFA-immunized mice were expanded ex vivo by stimulation with 5 μg/mL ovalbumin in the presence of mitomycin C-treated syngeneic splenocytes. Highly enriched ovalbumin-specific CD4^+^ T cells after three rounds of Ag stimulation were used in proliferation assays at 2 × 10^5^ cells/well and the addition of ovalbumin and 10^6^ cells/well mitomycin C-treated splenocytes.

For MLR to allogeneic cells, CD4^+^ T cells isolated from mice of C57BL/6 background were set up at 2 × 10^5^ cells/well and cocultured for 3 days with 2 × 10^6^ cells/well of mitomycin C-treated BALB/c allogeneic splenocytes. Proliferation of T cells was measured by overnight pulsing of cells with 1 μCi/well of ^3^H-thymidine (Perkin Elmer) followed by counting radioactivity of harvested cells in scintillant (Perkin Elmer) using a Topcount (Packard).

### Cytokine ELISA assays

Cytokines in T-cell culture supernatants were measured in ELISA assays using Ab pairs for IL-2, IL-4, and IFN-γ (BD Biosciences) and IL-17A ELISA kit (R&D) following the kit protocols.

### T-cell signaling assays

T cells were coated with 1 μg/mL of antimouse CD3ε (Biolegend) alone or in combination with anti-CD28 (Biolegend), followed by coupling with 1 μg/mL of goat antihamster IgG (Pierce). T cells before and after stimulation were lysed with NP-40 lysis buffer containing protease inhibitors (Invitrogen). Lysates were separated by electrophoresed and blotted onto polyvinylidene difluoride (PVDF) filters (Bio-Rad). AKT, ERK, and their phosphorylated forms, as well as PI3Kγ p110 were detected and quantitated in Western blots using specific antibodies (Cell Signaling Technology) following the protocols of reagent kits.

### T-cell phenotyping in flow cytometry

T cells were stained with fluorescent dye conjugated antibodies specific for different cell surface markers (eBioscience) according to standard staining procedures. Intracellular staining was performed by stimulating T cells for 4 h with a leukocyte activation cocktail containing GolgiPlug (BD Biosciences), followed by fixing with paraformaldehyde (Biolegend) and permeabilization with saponin buffer (Biolegend), and then staining with antibodies (eBioscience) according to kit protocols. Cell division was detected by staining T cells with 1 μM CFSE (Invitrogen) prior to activation or differentiation assays. Samples were acquired with FACSCalibur (BD Biosciences) and analyzed with FlowJo software (Tree Star).

### T-cell differentiation assays

Naive CD4^+^ T cells were activated with plate-bound anti-CD3 (1 μg/mL) and soluble anti-CD28 (2.5 μg/mL) under different polarizing conditions. Th1 differentiation was driven by 20 U/mL IL-12, 100 U/mL IL-2, and 10 μg/mL anti-IL-4. Th2 differentiation condition was set up with 200 U/mL IL-4, 100 U/mL IL-2, and 10 μg/mL each of anti-IL12 and anti-IFN-γ. Th17 polarization was prepared with 5 ng/mL TGF-β, 20 ng/mL IL-6, and 10 μg/mL each of anti-IL-4 and anti-IFN-γ (all reagents form ReproTech). Treg-cell differentiation condition was set by addition of 5 ng/mL TGF-β1 and 100 U/mL IL-2 in medium. After 6 days of differentiation, T cells were characterized by intracellular staining of cytokines and transcription factors as described in the earlier section on T-cell phenotyping.

Differentiation of Ag-specific T cells was performed with naïve CD4^+^ T cells from OT-II transgenic mice. Transgenic T cells were stimulated for 6 days with 0.5 μM ovalbumin peptide OVA323–339 (ISQAVHAAHAEINEAGR, Anaspec) in the presence of mitomycin C-treated splenocytes under Th1 or Th17 differentiation condition.

### Lipid raft immunofluorescence staining

CD4^+^ T cells were incubated with anti-CD3- or anti-CD3-coated plus anti-CD28-coated polystyrene beads (Spherotech) at 1:5 cell/bead ratio at 37°C for 30 min followed by cell attachment onto poly-l-lysine-coated slides (BD Pharmaceuticals). Cells on slides were treated with 3.7% paraformaldehyde in PBS for 10 min, permeabilized with 0.1% Triton X-100 in PBS for 10 min, and then blocked with 1% BSA in PBS for 30 minu. Cells were then incubated with FITC-conjugated cholera toxin B (Sigma) for 1 h, washed with PBS, and mounted onto slides with Vectashield containing 4′, 6′-diamidino-2-phenylindole (Vector Laboratories). Cells on slides were examined using a Leica SPE2 confocal microscope system with Zeiss 63 × 1.4 oil objectives and SlideBook 4.2 software (Intelligent Imaging Innovations). Cholera toxin B-stained lipid rafts at cell/bead contact areas were evaluated visually on ∼150 cell/bead conjugates.

### T-cell chemotaxis assays

Chemotaxis assays were performed in transwell plates with uncoated filters of 5 μm pore size (Corning Coastar). Splenocytes were placed at the upper chamber at 2 × 10^6^ cells/well and chemokine CCL3 (0.27 μg/mL), CCL19 (0.2 μg/mL), CCL21 (0.5 μg/mL), CXCL12 (3 μg/mL), or RANTES (0.1 μg/mL, R&D Systems) was added in the lower chamber. T cells entered in the lower chamber after 1 h of incubation were quantitated by acquisition with FACSCalibur.

### Ovalbumin immunization

PI3Kγ^KD/KD^ mice backcrossed to C57BL/6 background and WT C57BL/6 mice were immunized at tail base with 200 μg ovalbumin (Grade V, Sigma-Aldrich) emulsified with CFA (BD Diagnostics) at 1:1 volume ratio. For ovalbumin/alum immunization, BALB/cJ mice and PI3Kγ^KD/KD^ mice of BALB/cJ background were immunized at tail base with 50 μg ovalbumin (Grade V, Sigma-Aldrich)/alum mixture. Ovalbumin/alum mixture was prepared by mixing 200 μg/mL ovalbumin in PBS with 4% Imject Alum (Pierce) at 1:1 volume ratio and incubated for 1 h at room temperature. On day 10, postimmunization, mice were sacrificed and the draining inguinal lymph nodes were collected and cell suspensions were prepared for cell count, intracellular cytokine detection, and proliferation assays.

Draining lymph node cells from ovalbumin/CFA immunized mice were stimulated with ovalbumin ex vivo at 5 × 10^5^ cells/well for 3 days. Cell proliferation and cytokine production were measured with methodologies as described in T-cell proliferation section.

### Delayed-type hypersensitivity model

C57BL/6J mice and PI3Kγ^KD/KD^ mice were immunized at tail base with 100 μL of 1 mg/mL mBSA (Sigma) in 1:1 volume CFA emulsion (Difco; BD Diagnostics). On day 7 mice were challenged by injecting 50 μL of 180 μg/mL mBSA into left footpads and 50 μL of saline into right footpads as negative controls. Footpad thickness was measured with a caliper at 0, 6, 26, 29.5, and 48 h time points. On day 11, mice were sacrificed and inguinal nodes were collected to prepare cells for proliferation assays. Paws were collected and fixed in 10% formalin, decalcified with formic acid, processed, and longitudinally embedded in paraffin. Serial 5 μm sections were prepared and stained with H&E for histology evaluation. Tissue was scored for inflammation, edema, hemorrhage, and subcutaneous necrosis. Each parameter was scored as follows: 0 = Normal, 1 = Mild, 2 = Moderate, 3 = Moderately severe, 4 = Markedly Severe. A summary histological score for severity of inflammation was accounted as summary of all the parameters.

Draining inguinal lymph node cells from mBSA immunized mice were stimulated with different concentrations of mBSA at 5 × 10^5^ cells/well for 3 days. Cell proliferation and cytokine production was measured as described previously.

## Abbreviations

AKT: protein kinase B
FOXO: Forkhead box
GPCR: G protein-coupled receptor
KLF2: Krueppel-like factor 2
mBSA: methylated BSA
PIP3: phosphatidylinositol 3,4,5-triphosphate
PI3Kγ: phosphatidylinositol-3-kinase gamma
PI3Kγ^KD/KD^: PI3Kγ kinase-dead knock-in
PVDF: polyvinylidene difluoride

## Supporting Information

As a service to our authors and readers, this journal provides supporting information supplied by the authors. Such materials are peer reviewed and may be re-organized for online delivery, but are not copy-edited or typeset. Technical support issues arising from supporting information (other than missing files) should be addressed to the authors.

**Figure S1**. Normal expression level of mutated PI3Kγ protein in CD4^+^ T cells of PI3Kγ^KD/KD^ mice. **Figure S2**. CD4^+^ T-cell gating strategy in FACS analysis. Data shown were splenocytes from wild-type mice.
