# Supplementary material for: PI3Kγ kinase activity is required for optimal T-cell activation and differentiation
Source: Eur J Immunol. 2013 Oct 9;43(12):3183–96. doi: 10.1002/eji.201343812 (PMC4209804; doi:10.1002/eji.201343812)
Supplement: Supplementary file 1 [file eji0043-3183-sd1.pdf]

SUPPORTING INFORMATION FIGURE 1. Normal expression level of mutated PI3Kγ protein in $CD4^+$ T cells of $PI3K\gamma^{KD/KD}$ mice. PI3Kγ catalytic subunit p110γ in wild-type (WT) and $PI3K\gamma^{KD/KD}$ (KD) $CD4^+$ T cells were identified with a specific antibody in immunoblots as described in Materials and Methods.

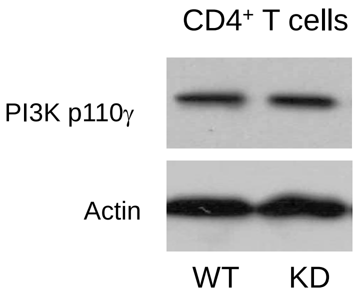

Supplemental Figure 1

SUPPORTING INFORMATION FIGURE 2. $CD4^+$ T-cell gating strategy in FACS analysis. Data shown were splenocytes from wild-type mice. Live cells in splenocytes were gated in the FSC/SSC plot as indicated in the circled area. CD4 T cells in the selected live cell population were gated in the CD4/FSC plot as indicated in the circled area. Gated $CD4^+$ T-cell populations were used in FACS analysis to monitor expression of cell surface markers and intracellular cytokines or transcription factors.

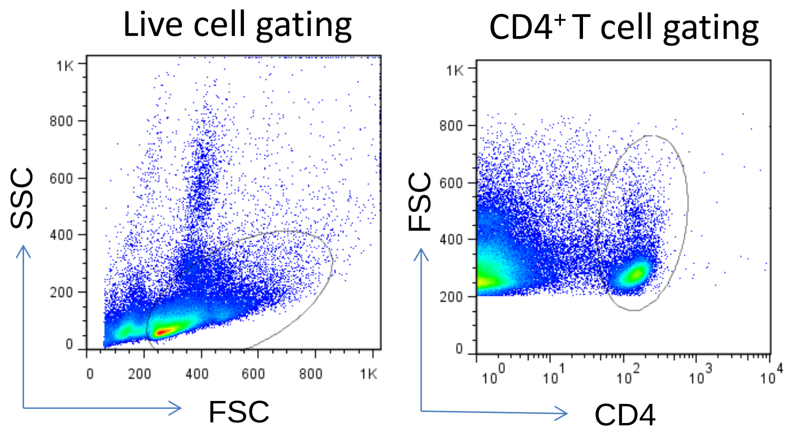

Supplemental Figure 2
